# Impact of Education on Weight in Newly Diagnosed Type 2 Diabetes: Every Little Bit Helps

**DOI:** 10.1371/journal.pone.0129348

**Published:** 2015-06-08

**Authors:** Kristen M. J. Azar, Sukyung Chung, Elsie J. Wang, Beinan Zhao, Randolph B. Linde, Janet Lederer, Latha P. Palaniappan

**Affiliations:** 1 Palo Alto Medical Foundation Research Institute, Palo Alto, California, United States of America; 2 Palo Alto Medical Foundation, Palo Alto, California, United States of America; 3 Stanford University School of Medicine, Palo Alto, California, United States of America; Weill Cornell Medical College in Qatar, QATAR

## Abstract

**Aims:**

Highly structured, intensive behavioral lifestyle interventions have been shown to be efficacious in research settings for type 2 diabetes management and weight loss. We sought to evaluate the benefit of participation in more limited counseling and/or education among individuals with newly diagnosed type 2 diabetes in more modest real-world clinical settings.

**Methods:**

Electronic Health Records of newly diagnosed type 2 diabetes patients age 35–74 from a large ambulatory group practice were analyzed (n = 1,314). We examined participation in clinic-based lifestyle counseling/education and subsequent weight loss.

**Results:**

Of the total cohort, 599 (45.6%) patients received counseling/education with (26.2%) and without (19.4%) medication, 298(22.7%) patients received a prescription for medication alone, and 417(31.7%) patients were only monitored. On average, those who participated in counseling/education attended 2.5 sessions (approximately 2–3 hours). The average weight loss of patients who received counseling/education alone during the follow-up period (up to three years post-exposure to participation) was 6.3 lbs. (3.3% of body weight), and, if received with medication prescription, 8.1lbs. (4.0% of body weight) (all at P<0.001). The weight loss associated with medication was only 3.5 lbs. (P<0.001). No significant weight change was observed in the monitoring only group.

**Conclusions:**

While efforts to improve both the short-term and long-term effectiveness of behavioral lifestyle interventions in real-world settings are ongoing, it is important for clinicians to continue to utilize less intensive, existing resources. Even relatively small “doses” of health education may help in promoting weight loss and may potentially reduce cardiometabolic risk.

## Introduction

Guidelines for the treatment of type 2 diabetes recommend early initiation of therapy, with lifestyle counseling and medication offered concomitantly.[1, 2] Education is critical to helping individuals implement and sustain healthy lifestyle changes, self-manage type 2 diabetes, adhere to prescribed treatments, and utilize appropriate health services.[3] Yet, studies within the U.S. and Canada have revealed low rates of utilization of existing type 2 diabetes education and self-management programs.[3–6]

Highly structured, intensive behavioral lifestyle interventions, such as the Look AHEAD Program, have been shown to improve type 2 diabetes control and cardiovascular disease (CVD) risk factors, and reduce medication use primarily by promoting weight loss. [7–10] Intensive lifestyle interventions have also been associated with substantial remission rates.[9] Much work remains to translate such highly structured and resource-intensive interventions into real-world clinical and/or community settings [11, 12] in a manner that produces a comparable effect size in clinical outcomes and is sustainable.

In clinical practice, there is wide variation in lifestyle intervention programs offered as an initial treatment to patients with newly diagnosed type 2 diabetes, and the amount of education tends to be relatively small.[13] Treatment components may include informal counseling by a clinician, formal referral to more intensive education programs or specialists, or any combination of those elements, in conjunction with prescription of medication. Little is known about the effectiveness of existing clinic-based behavioral programs, and it is unclear whether small exposure to lifestyle intervention results in clinically meaningful benefit.

We sought to examine the effectiveness of clinic-based behavioral lifestyle (diet and/or physical activity) counseling/education interventions in promoting weight loss among individuals with newly diagnosed type 2 diabetes. We used observational data available through electronic health records (EHR) in a large, mixed-payer, ambulatory care organization to understand the effectiveness of intervention programs offered in a real world setting.

## Materials and Methods

### Study setting

Demographic and clinical data of study participants were extracted from the EHRs in a large multi-specialty, mixed-payer, outpatient, group practice organization in northern California. The demographic characteristics of the patient population are similar to that of residents in the surrounding service area.[14]

### Ethics statement

All datasets analyzed by the research team were HIPAA de-identified. The study received approval from the Palo Alto Medical Foundation Institutional Review Board on April 2, 2009. Our IRB waived the requirement of an informed consent.

### Subjects

We defined incident type 2 diabetes cases as patients age 35–74 years who had the *first* confirmatory evidence of type 2 diabetes during the surveillance period: January 1, 2007 to June 30, 2010 (n = 2,577). Confirmatory evidence of diabetes was defined as (i) two abnormal laboratory values (hemoglobin A1c [HbA1c], fasting glucose, random glucose, and oral glucose tolerance tests)[15] or (ii) physician diagnosis of type 2 diabetes (ICD-9 codes 250.00–250.92) as noted in the EHR.

Patients who were (i) pregnant during the follow-up period (n = 4); or (ii) had serious clinical conditions including cancer (n = 379), liver (n = 131), and kidney disease (n = 114) were excluded, given that weight loss in these patients is generally not recommended. We further excluded patients who had no clinic visit that addressed type 2 diabetes (identified with visit diagnosis) within the 12 months following initial evidence of type 2 diabetes (n = 360). Among the eligible patients with incident type 2 diabetes (n = 1,589), 275 patients were excluded because they did not have a weight measurement during the observation period, leaving 1,314 patients included in the analysis.

### Treatment approaches

Treatment was identified based on clinical activities within the 12 months following a new diagnosis of type 2 diabetes. We examined the effectiveness of four treatment options: formal behavioral lifestyle counseling/education (hereafter referred to as counseling/education) only; diabetes medication prescription only; both counseling/education and prescription of medication; and monitoring only. Monitoring only is not a lack of diabetes care, but rather “active monitoring” as patients in this group made follow-up visits addressing diabetes.

Participation in counseling/education was identified if a patient participated in individual counseling with a registered dietitian or nutritionist or a group-based class focused on behavioral lifestyle modification, weight management, and/or type 2 diabetes. Counseling/education offerings varied in curriculum, session length, group size, and number of consecutive sessions offered. Group-based classes offer care for small groups of patients (6–12 per group) through semi-structured education and group support specifically for chronic disease management. Individual counseling includes a referral to a registered dietician or nutritionist who provides individualized, in-person, one-on-one education. The length of each session, either group class or individual counseling, ranged 60–90 minutes. While educational offerings differed in curriculum and format, all focused on guideline-adherent healthy diet and physical activity. More information on the content of these classes can be found at: http://www.pamf.org/healtheducation/classes/. The vast majority of education/counseling sessions the patients included in this study cohort participated in were either group classes (71%) or individual counseling (19%) aimed specifically at diabetes education (e.g. Diabetes Education; Living Well with Diabetes; Introduction to Diabetes; Healthy Eating for Type 2 Diabetes; Diabetes Shared Medical Appointment). Approximately 3% participated in either individual counseling or a group class focused on pre-diabetes and the remaining 7% participated in either group or individual session(s) providing general dietary and nutrition guidance. Insurance coverage varied. Classes and individual consultations were offered by trained clinical professionals (i.e. dietitian).

### Analytical approach

A difference-in-differences approach was used to estimate pre-post weight change associated with treatment. Body weights measured during the study period, ranging from 48 months prior-to and up-to 36 months following initial diagnosis (i.e., first confirmatory evidence of type 2 diabetes), were included in the analyses. When a patient received multiple counseling/education sessions, the period after the last counseling/education session was considered as the post-counseling/education period. We focused on the treatment received within the first 12 months of initial diagnosis, so if a patient received initial counseling/education or medication prescription *after* the 12 months following initial diagnosis, the patient was grouped into “monitoring only” group, and weight measurements before the initial treatment were included. On average, a patient had 12 weight measures, 7 at or prior to initial diagnosis and 5 post-diagnosis.

The difference-in-differences estimates were drawn from multivariate models where the coefficients for main treatment indicators (i.e., an interaction term of “treatment choice” and “post treatment period”) represent pre-post weight change associated with each treatment. The multivariate models incorporated propensity score adjustment. One important consideration of using observational data, rather than a randomized controlled trial, is potential selection bias. That is, a patients’ weight loss may not be entirely attributable to the treatment because the participants may be more motivated to lose weight regardless of the intervention. To mitigate this concern, we estimate propensity score, defined as the likelihood of each patient receiving treatment counseling/education, and included it as a covariate. Similarly, the propensity score of medication prescription was also estimated and included as a separate covariate. The propensity score was estimated with a logistic model with all the covariates in the main regression model plus weight at initial diagnosis and physician’s referral to a counseling/education session. The regression results of propensity score estimation are available in S1 Appendix.

**Fig 1:**
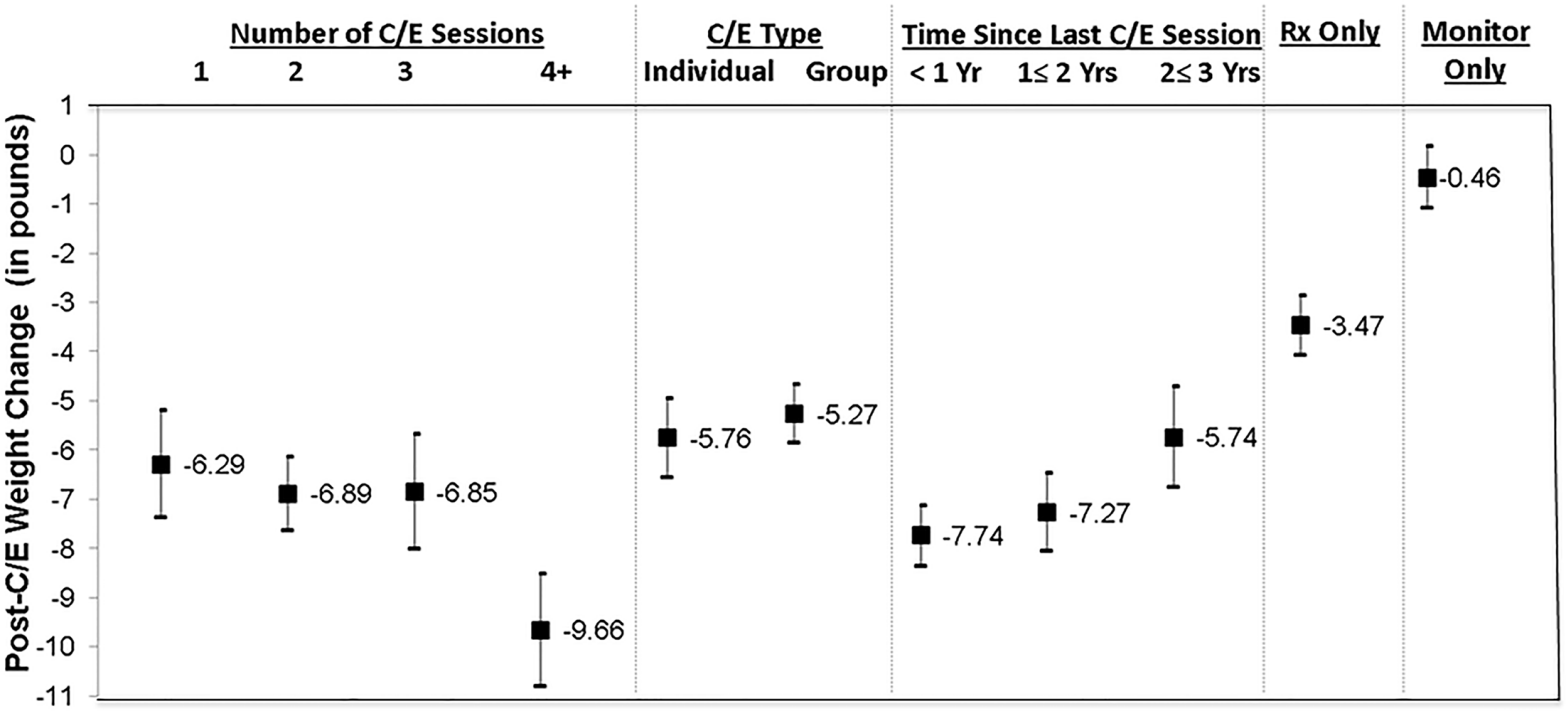
Weight Change Associated with Doses and Types of Counseling/Education (C/E)^(a)(b)^. (a) Classification of C/E type is based on those who received any C/E, regardless medication prescription or not. (b) Estimates are from propensity-score adjusted individual random effects models (Number of observations = 16,064; Number of patients = 1,314). Coefficient and 95% Confidence Intervals are presented above. In all the models, patient clinical and demographic characteristics (any medication prescription during 12 months follow-up, any education within 1 year prior to baseline, fasting blood glucose level at baseline, height, age, sex, race/ethnicity, comorbidities (cardiovascular disease, dyslipidemia, hypertension), smoking status, frequency of primary care visits during 12 months follow-up, any endocrinologist visit during 12 months follow-up) were included. For the estimation of propensity score, additional variables indicating physician order of education/counseling during 12 months follow-up and initial body weight was included.

In addition to main treatment effects, we examined dose-response relationship, using four categories of exposure: 1, 2, 3, and 4 or more sessions. We also examined the difference in the effect size between group-based and individual counseling/education. Further, we compared weight trajectory for a period of up to three years post type 2 diabetes diagnoses in each treatment group (Fig 1).

In addition to propensity scores, potential confounders of the relationship between treatment choice and weight outcome were included as covariates in multivariate models. Covariates included were indicators of treatment choice, clinical risk factors at initial diagnosis (fasting blood glucose ≥130 mg/dL, height, cardiovascular disease, hyperlipidemia, hypertension), service use (frequency of primary care visits and endocrinologist during the 12 months of follow-up, counseling/education session addressing lifestyle intervention received prior to initial diagnosis), and demographics (age, sex, race/ethnicity, insurance type). Individual random effects were included to take into account clustering of multiple weight measures of a patient. The use of individual random effects, rather than individual fixed effects, was chosen based on the Hausman test (*P*>0.1 in all models).

## Results

### Baseline demographic and clinical characteristics

The average age of patients was 54, and 41% were female (Table 1). The majority of patients were non-Hispanic white (49.7%) followed by Asian (36.8%). About half (46.0%) had a baseline fasting blood glucose level ≥130 mg/dL. The average weight was 198.0 lbs. prior to baseline (diagnosis) and was 195.0 lbs. at follow-up.

**Table 1:** Comparison of Clinical Characteristics at Initial Type 2 diabetes Diagnosis, by Treatment Type during 12 Months Post-Diagnosis Follow-up.

Variable (range)	OverallN = 1,314	C/E OnlyN = 255 (19.4%)	Medication Prescription onlyN = 298 (22.7%)	C/E and Medication N = 344(26.2%)	Monitoring onlyN = 417 (31.7%)	P value
**Clinical risk factors**						
Fasting glucose ≥130mg/dL at diagnosis	46.0%	110 (43.1%)	161 (54.0%)	204 (59.3%)	130 (31.2%)	<0.001
Weight at diagnosis (lb.) ^(a)^	194.2 [47.6]	192.2 [43.2]	200.0 [52.6]	202.4 [50.0]	184.7 [42.5]	<0.001
Height at diagnosis (inch) ^(a)^	66.5 [4.0]	66.9 [4.1]	66.8 [3.9]	66.5 [4.0]	66.1 [3.9]	0.03
BMI at diagnosis (kg/m^2^)	30.7 [6.3]	30.0 [5.4]	31.4 [7.0]	32.1 [6.8]	29.5 [5.5]	<0.001
Average weight (lb.) prior to treatment ^(b)^	197.7 [48.7]	196.0 [44.8]	201.8 [51.1]	206.9 [53.1]	188.1 [43.5]	<0.001
Average weight (lb.) post treatment ^(c)^	194.0 [49.4]	190.2 [44.5]	198.9 [52.8]	201.0 [52.7]	187 [45.6]	<0.001
**Demographics**						
Age	53.8 [10.2]	55.3 [9.9]	52.3 [9.6]	52.6 [10.2]	55.0 [10.6]	0.02
Female	40.6%	38.0%	34.6%	43.9%	43.9%	0.03
Race/ethnicity						
Non-Hispanic white	49.8%	54.5%	50.7%	52.3%	44.1%	0.04
Asian	36.8%	33.3%	37.2%	30.2%	43.9%	<0.001
Black	2.1%	1.6%	1.3%	2.0%	3.1%	0.36
Latino	11.3%	10.6%	10.7%	15.4%	8.9%	0.04
**Service use**						
Any endocrinology visit	5.4%	3.1%	5.4%	8.4%	4.3%	0.02
Primary care visits (range 0–95)	9.8 [10.7]	9.3 [11.2]	9.2 [9.1]	11.1 [11.2]	9.5 [10.9]	0.08
Any education, 12 months *prior to* type 2 diabetes diagnosis	4.6%	4.7%	3.0%)	3.8%	6.5%	0.14

Data are the Mean [SD]; frequency %.

(a) Closest available weight measurement to baseline/diagnosis of type 2 diabetes was used.

(b) Up to 48 months pre diagnosis of type 2 diabetes.

(c) Up to 36 months post diagnosis of type 2 diabetes.

Almost half (45.6%) received counseling/education treatment, some (26.2%) with and others (19.4%) without medication prescription. About a quarter (22.7%) received a prescription of diabetes medication. The remainder (31.7%) received active monitoring only. For those who received medication prescription, most (90.1%) received metformin (not reported in the table). Other prescribed medications included Glyburide (5%), Glipizide (2.5%), Pioglitazone (1.0%), as well as Glimepiride, Sitagliptin, Repaglinide, Rosiglitazone (< 1.0% respectively). No patients in our cohort were prescribed insulin. Patients who received medication prescription (with or without counseling/education) were likely to have a higher level of fasting blood glucose at diagnosis and body weight both prior to and post diagnosis (all at *P*<0.001). Demographic and service use characteristics were similar across treatment groups; one exception is that Asian patients were more likely to receive monitoring only (P<0.001).

Among those who participated in any counseling/education, the number of sessions attended within 12 months of diagnosis ranged from 1 to 11, but most received 2 sessions (mean 2.5; median 2), 40% received individual counseling, 79% received a group-based class, 19% received both, and treatment was initiated on average within 59 days of initial diagnosis (Table 2).

**Table 2:** Frequency of Counseling/Education Sessions.

	Mean [SD] or N (frequency %)
Any counseling/education session attended ^(a)^	599 (45.6%)
Among patients who attended any, frequency of counseling/education sessions	
Counseling/education classes (range 1–11)	2.5 [1.4]
Any individual counseling	242 (40.4%)
If any, individual counseling (range 1–7)	1.6 [1.0]
Any group counseling/class	470 (78.5%)
If any, group counseling/class (range 1–6)	2.4 [1.2]
Both individual and group counseling/class	113 (18.9%)
Days to the 1^st^ session since initial diagnosis (range 0–356)	59.0 [75.3]

(a) During the follow-up period of 12 months post-diagnosis of type 2 diabetes.

### Weight change with counseling/education, medication prescription, both treatments, and monitoring only

Multivariate results, after adjusting for all covariates and propensity scores, show that average weight loss associated with counseling/education only (no medication) within the first year of diagnosis was 6.3 lbs. or 3.3% of body weight at initial diagnosis (Table 3). Weight loss associated with medication prescription only (no counseling/education) was 3.5 lbs. or 1.7% of initial weight. Average weight loss among those who received both counseling/education and medication prescription was 8.1 lbs. or 4.0% of body weight (all at *P*<0.001). No significant weight change was observed in the monitoring only group. Regression results of all included covariates are available in S2 Appendix.

**Table 3:** Effect of Counseling/Education (C/E) on Weight (a).

	Coefficient (SE)
C/E only * post-treatment (i.e., post-treatment weight *change* with C/E only)	-6.26** (0.39)
Medication only * post-treatment	-3.47** (0.31)
C/E and Medication * post-treatment	-8.05** (0.33)
Monitoring only * post-diagnosis	-0.46 (0.32)

** p<0.001

Number of observations: 16,064; Number of patients = 1,314.

(a) In all the models, propensity scores (of receiving C/E and receiving medication prescription), indicators of treatment choice, and patient clinical and demographic characteristics (any medication prescription during 12 months follow-up, any education within 1 year prior to baseline, fasting blood glucose level at baseline, height, age, sex, race/ethnicity, comorbidities (cardiovascular disease, dyslipidemia, hypertension), smoking status, frequency of primary care visits during 12 months follow-up, any endocrinologist visit during 12 months follow-up) were included. For the estimation of propensity score, additional variables indicating physician order of education/counseling during 12 months follow-up and initial body weight was included.

### Effect of counseling/education on weight by dose, type and time elapsed

People lost more weight as the frequency of counseling/education increased, but the relationship is not linear. Participation in one to three sessions of counseling/education (with or without medication prescription) was associated with weight loss of 6.3 to 6.9 lbs., which corresponds to 3.2 to 3.5% of body weight. Four or more counseling/education sessions was associated with substantially higher weight loss, 9.7 lbs. (4.8% of body weight) (all at *P*< 0.001) (Fig 1). There was no significant incremental weight loss after four sessions (data not shown).

By type of counseling/education, those who received individual counseling lost 5.8 lbs. (2.9% of body weight) and those who received group-based class lost 5.3 lbs. (2.7% of body weight) (both significantly different from referent group at *P*< 0.001).

The effect of counseling/education treatment lasted several years (we observed up to three years) although effect size reduced as time elapsed since the exposure to last counseling/education. On average, body weight was the lowest in the first year of treatment (7.7 lbs. less than prior to treatment) and then gradually increased (-7.3 lbs. in the second year post-treatment; -5.7 lbs. in the third year post-treatment) (all at P<0.001). The regression results of all covariates are available in S2 Appendix.

## Discussion

Our aim for this study was to investigate the effectiveness of counseling/education in a real practice setting among individuals with newly diagnosed type 2 diabetes. The findings of our study suggest that even a small “dose” of formal behavioral lifestyle counseling and/or education (i.e., attending one session) offered at outpatient clinics can be beneficial and promotes modest weight loss.

Modest weight loss (3–10% reduction in total body weight)[16] for individuals who are overweight or obese has been shown to produce health benefits such as improvement in blood pressure, cholesterol and dysglycemia.[16–21] Clinically significant weight loss of 3–10% reduction in body weight is important for several reasons beyond the health benefits conferred and potential reduction in cardiometabolic risk. First, given that more than one-third (35.7%) of U.S. adults are obese[22] and individuals in the U.S. gain approximately 1 pound per year on average,[23] any weight reduction changes the default trajectory of weight gain and may be helpful in reducing cardiometabolic risk. Second, modest weight loss can serve as motivation for continued engagement and participation in treatment by increasing confidence and self-efficacy.[24] This may in part be due to a shift from a perceived external locus of control to an internal locus,[24] whereby individuals begin to feel more in control and empowered to influence their own health outcomes and quality of life.

The effects on weight reduction were observed in spite of minimal participation in counseling/education. Of those who attended individual counseling sessions, a vast majority attended only one (63%) or two (24%) sessions. Similar findings were observed for those who attended group-based classes, where a majority attended one or two sessions (15% and 60%, respectively). In a previous study among overweight individuals at the same healthcare institution,[25] participants in a group-based, physician-led class for weight management attended 1–2 sessions and lost on average 1.0% of their baseline weight over the course of a year. In comparison, individuals who did not participate showed a 0.8% increase in body weight from baseline.

Our findings suggest that a “dose-response” relationship may exist where effect size markedly increased when patients attended 4 sessions versus 1–3 sessions, then plateaued after 4 sessions. There may be some value in determining optimal “dose” at which clinically significant benefit is observed. For example, the Look AHEAD trial[7, 26] involved an intensive coach-led lifestyle intervention consisting of a combination of weekly group meetings and individual counseling sessions over the course of 6 months.[7, 26] While the intervention demonstrated significant clinical improvement among the participants in glycosylated hemoglobin level, greater likelihood of partial remission of type 2 diabetes, increased quality of life, and greater weight loss as compared to the control group,[9, 26–28] the program is highly structured and extremely resource intensive in its intended formulation. Barriers to effectiveness of translating highly structured research interventions in real-world settings include reduced access and adherence among participants due to competing priorities. In addition to amount of contact with health care providers, several other factors were associated with weight loss including high baseline fasting blood glucose values and history of CVD and dyslipidemia. These additional comorbidities may have increased patient motivation to seek help and participate in counseling and/or education.

Innovative approaches are needed to improve access and convenience, to ensure that individuals participate at optimal intensity to achieve realistic and sustainable outcomes.[29] Group-based interventions may provide some of these elements. Given that both individual and group counseling/education treatments resulted in similar degree of weight loss in our study, group approaches are more cost-effective[30] and beneficial in sustaining weight loss, possibly from the added benefit of social support.[30] The inclusion of a social support component in weight management interventions has been recognized as a key factor in weight loss success.[31, 32] These findings are consistent with other studies where group interventions have been shown to be a cost-effective type 2 diabetes self-management intervention in that it helps to improve the participants’ glycemic control and promote weight loss.[27, 33, 34]

Several limitations, mostly due to the defined scope of our study, merit discussion. First, we cannot rule out the possibility of selection bias (i.e., patients who received counseling/education may be more motivated to lose weight than those who did not). While we attempted to adjust for observed differences among treatment groups in the multivariate analyses by using propensity score and controlling for potential confounders, the effect of counseling/education may be at least partially driven by “motivation” among those who chose to attend counseling/education independent of intervention. Particularly in our dose response analysis, while 79% of patient within the C/E groups participated in at least 2 sessions, only 20% of patients within the C/E groups participated in >3 sessions, and these probably represented the most motivated patients. Second, adherence to medication was not examined, as we did not have access to prescription fill data. Therefore, actual rate of medication use by patients may be lower, and the effect of medication treatment, when adhered as prescribed, would be larger. Counseling/education dose, however, was based on attendance. Third, as our study focused on treatment options in clinical settings, we did not examine patient-initiated lifestyle modifications outside of the clinical care realm.

In conclusion, while efforts to improve both short-term and long-term effectiveness of behavioral lifestyle interventions in real-world settings are ongoing, it is important for clinicians to continue to utilize existing resources because even a small dose of education helps in promoting weight loss and risk reduction. Individuals who are newly diagnosed with type 2 diabetes may be particularly receptive to learning about how to self-manage their type 2 diabetes and even potentially partially reverse it through behavioral lifestyle change. It is important for clinicians to take advantage of this “teachable moment” and refer individuals for formal counseling with a dietician or a structured class as deemed appropriate to gain further understanding of how their current behaviors may impact their type 2 diabetes status.

## Supporting Information

S1 Appendix First stage – prediction of likelihood of (A) any education/counseling (C/E) and (B) any medication prescription treatments. Odds ratio (95 CI) presented; Number of observations = 1,314. *** p<0.001, ** p<0.01, * p<0.05.^1^ T2DM = type 2 diabetes mellitus. ^2^ Height centered (height—average height). ^3^ Age centered (age–average age).
(PDF)

S2 Appendix Effect of Treatments (varying specifications) on Weight, Multivariate Analysis Results. Coefficient (Standard Error) presented; Number of observations: 16,064.C/E = Counseling/Education; T2DM = type 2 diabetes mellitus. Number of observations: 16,064; Number of patients = 1,314. *** p<0.001, ** p<0.01, * p<0.05.
(PDF)
