# Supplementary material for: Impact of Education on Weight in Newly Diagnosed Type 2 Diabetes: Every Little Bit Helps
Source: PLoS One. 2015 Jun 8;10(6):e0129348. doi: 10.1371/journal.pone.0129348 (PMC4459994; doi:10.1371/journal.pone.0129348)
Supplement: S1 Appendix — Odds ratio (95 CI) presented; Number of observations = 1,314. *** p<0.001, ** p<0.01, * p<0.05.1 T2DM = type 2 diabetes mellitus. 2 Height centered (height—average height). 3 Age centered (age–average age). (PDF) [file pone.0129348.s001.pdf]

Appendix 1.

First stage – prediction of likelihood of (A) any education/counseling (C/E) and (B) any medication prescription treatments

Variables	(A) Counseling/education		(B) Medication prescription	
	OR	95 CI of OR	OR	95 CI of OR
Order for C/E within 12 months post T2DM diagnosis	2.51***	(1.91 - 3.30)	1.87***	(1.42 - 2.47)
Baseline weight	1.00	(1.00 - 1.00)	1.01***	(1.00 - 1.01)
C/E within 12 months prior to T2DM diagnosis	0.95	(0.55 - 1.63)	0.70	(0.40 - 1.25)
Primary care visit within 12 months post T2DM diagnosis	0.99	(0.98 - 1.00)	1.00	(0.98 - 1.01)
Endocrinology visit within 12 months post T2DM diagnosis	1.12	(0.68 - 1.85)	1.55	(0.92 - 2.62)
Height[2]	1.03	(0.98 - 1.08)	0.94*	(0.90 - 0.99)
Fasting blood glucose ≥ 130 mg/dL	1.57***	(1.25 - 1.98)	2.23***	(1.77 - 2.82)
Age[3]	1.00	(0.99 - 1.02)	0.98***	(0.96 - 0.99)
Female	1.30	(0.93 - 1.81)	0.96	(0.68 - 1.34)
Asian race	0.81	(0.60 - 1.09)	0.85	(0.63 - 1.15)
Black race	0.76	(0.34 - 1.69)	0.74	(0.33 - 1.68)
Latino ethnicity	1.34	(0.91 - 1.96)	1.14	(0.77 - 1.70)
Ever had cardiovascular disease	0.73	(0.48 - 1.11)	0.86	(0.57 - 1.31)
Ever had dyslipidemia	0.95	(0.74 - 1.21)	1.00	(0.78 - 1.28)
Ever had hypertension	1.12	(0.87 - 1.45)	1.12	(0.86 - 1.45)
Currently smokes	0.73	(0.22 - 2.36)	0.26	(0.067 - 1.04)
Constant	0.48*	(0.24 - 0.96)	0.16***	(0.079 - 0.34)

Odds ratio (95 CI) presented; Number of observations=1,314
*** p<0.001, ** p<0.01, * p<0.05
[1] T2DM = type 2 diabetes mellitus
[2] Height centered (height - average height)
[3] Age centered (age – average age)
