# Supplementary material for: Impact of Education on Weight in Newly Diagnosed Type 2 Diabetes: Every Little Bit Helps
Source: PLoS One. 2015 Jun 8;10(6):e0129348. doi: 10.1371/journal.pone.0129348 (PMC4459994; doi:10.1371/journal.pone.0129348)
Supplement: S2 Appendix — Coefficient (Standard Error) presented; Number of observations: 16,064.C/E = Counseling/Education; T2DM = type 2 diabetes mellitus. Number of observations: 16,064; Number of patients = 1,314. *** p<0.001, ** p<0.01, * p<0.05. (PDF) [file pone.0129348.s002.pdf]

Appendix 2. Effect of Treatments (varying specifications) on Weight, Multivariate Analysis Results

VARIABLES	Dependent variable: body weight<br>Coeff (SE) presented			
	Model 1	Model 2	Model 3	Model 4
Post-treatment, by treatment choice (ref=prior to treatment or diagnosis, of each treatment group)				
C/E only	-6.26***			
	(0.39)			
Medication prescription only	-3.47***	-3.43***	-3.36***	-3.40***
	(0.31)	(0.31)	(0.31)	(0.31)
Both C/E & medication prescription	-8.05***			
	(0.33)			
Monitoring only	-0.46	-0.46	-0.46	-0.46
	(0.32)	(0.32)	(0.32)	(0.32)
Post treatment effect, by type/dose of C/E				
1 C/E session		-6.29***		
		(0.55)		
2 C/E sessions		-6.89***		
		(0.39)		
3 C/E sessions		-6.85***		
		(0.59)		
4 or more C/E sessions		-9.66***		
		(0.57)		
Individual Counseling			-5.76***	
			(0.41)	
Group-based class			-5.27***	
			(0.31)	
1 year post C/E				-7.74***
				(0.31)
2 years post C/E				-7.27***
				(0.40)
3 years post C/E				-5.74***
				(0.52)
Treatment choice				
C/E only	-0.22	-0.016	-0.15	0.072
	(1.25)	(1.25)	(1.25)	(1.25)
Medication prescription only	-0.35	-0.36	-0.37	-0.37
	(1.21)	(1.21)	(1.21)	(1.21)
Both C/E & medication prescription	1.38	1.11	0.84	1.13
	(1.21)	(1.20)	(1.20)	(1.21)
C/E within 12 months prior to T2DM diagnosis	49.2***	49.1***	49.1***	49.1***
	(2.12)	(2.12)	(2.12)	(2.12)
Primary care visit within 12 months post T2DM diagnosis	-0.21***	-0.21***	-0.21***	-0.21***
	(0.043)	(0.043)	(0.043)	(0.043)
Endocrinology visit within 12 months post T2DM diagnosis	-52.3***	-52.2***	-52.1***	-52.2***
	(2.01)	(2.01)	(2.01)	(2.01)
Height	11.9***	11.9***	11.9***	11.9***
	(0.17)	(0.17)	(0.17)	(0.17)
Fasting blood glucose ≥ 130 mg/dL	-77.7***	-77.7***	-77.6***	-77.7***

	(1.47)	(1.47)	(1.47)	(1.47)
Age	3.57***	3.57***	3.57***	3.57***
	(0.078)	(0.078)	(0.078)	(0.078)
Female	35.9***	35.9***	35.9***	35.9***
	(1.29)	(1.29)	(1.29)	(1.29)
Asian race	-1.14	-1.13	-1.12	-1.13
	(1.17)	(1.17)	(1.17)	(1.17)
Black race	14.9***	14.9***	15.0***	14.9***
	(3.03)	(3.04)	(3.04)	(3.04)
Latino ethnicity	9.19***	9.17***	9.28***	9.20***
	(1.46)	(1.46)	(1.46)	(1.46)
Ever had cardiovascular disease	-7.44***	-7.45***	-7.53***	-7.48***
	(1.59)	(1.59)	(1.59)	(1.59)
Ever had dyslipidemia	-4.03***	-4.04***	-4.04***	-4.03***
	(0.92)	(0.92)	(0.92)	(0.92)
Ever had hypertension	-0.38	-0.37	-0.44	-0.38
	(0.98)	(0.98)	(0.98)	(0.98)
Currently smoke	159***	159***	159***	160***
	(4.66)	(4.66)	(4.66)	(4.66)
Propensity score of receiving C/E (0-1)	-427***	-426***	-427***	-427***
	(7.52)	(7.52)	(7.53)	(7.53)
Propensity score of receiving medication prescription (0-1)	662***	662***	662***	662***
	(8.80)	(8.81)	(8.81)	(8.81)
Constant	91.9***	91.7***	92.0***	91.9***
	(2.52)	(2.52)	(2.52)	(2.52)

Coefficient (Standard Error) presented; Number of observations: 16,064
C/E = Counseling/Education; T2DM = type 2 diabetes mellitus
Number of observations: 16,064; Number of patients=1,314
*** $p<0.001$, ** $p<0.01$, * $p<0.05$
